# Identification of PblB mediating galactose-specific adhesion in a successful *Streptococcus pneumoniae* clone

**DOI:** 10.1038/srep12265

**Published:** 2015-07-21

**Authors:** Yu-Chia Hsieh, Tzu-Lung Lin, Che-Ming Lin, Jin-Town Wang

**Affiliations:** 1 Department of Pediatrics, Chang Gung Children’s Hospital, Chang Gung Memorial Hospital, Chang Gung University, College of Medicine, Taoyuan, Taiwan; 2 Departments of Microbiology, National Taiwan University College of Medicine, Taipei, Taiwan; 3 Departments of Internal Medicine, National Taiwan University Hospital, Taipei, Taiwan

## Abstract

The pneumococcal genome is variable and there are minimal data on the influence of the accessory genome on phenotype. Pneumococcal serotype 14 sequence type (ST) 46 had been the most prevalent clone causing pneumonia in children in Taiwan. A microarray was constructed using the genomic DNA of a clinical strain (NTUH-P15) of serotype 14 ST46. Using DNA hybridization, genomic variations in NTUH-P15 were compared to those of 3 control strains. Microarray analysis identified 7 genomic regions that had significant increases in hybridization signals in the NTUH-P15 strain compared to control strains. One of these regions encoded PblB, a phage-encoded virulence factor implicated (in *Streptococcus mitis*) in infective endocarditis. The isogenic *pblB* mutant decreased adherence to A549 human lung epithelial cell compared to wild-type NTUH-P15 strain (P = 0.01). Complementation with *pblB* restored the adherence. PblB is predicted to contain a galactose-binding domain-like region. Preincubation of NTUH-P15 with D-galactose resulted in decreases of adherence to A549 cell in a dose-dependent manner. Challenge of mice with NTUH-P15, isogenic *pblB* mutant and *pblB* complementation strains determined that PblB was required for bacterial persistence in the nasopharynx and lung. PblB, as an adhesin mediating the galactose-specific adhesion activity of pneumococci, promote pneumococcal clonal success.

*Streptococcus pneumoniae*, a frequent colonizer of the nasopharynx of healthy children, is a major cause of invasive disease in children. In Taiwan, pneumonia was the most common disease in children^1^. Before the introduction of 7-valent pneumococcal conjugate vaccine in 2005, serotype 14 was the predominant type causing pneumonia. Among strains of serotype 14 causing pneumonia, sequence type (ST) 46 was the most prevalent clone^2^,^3^. The serotype 14 ST 46 clone accounted for 15% to 35% of the strains causing culture-confirmed pneumococcal pneumonia among children in Taiwan^2^,^4^.

It is known that certain clones of *S. pneumoniae* succeessfully disseminate in some regions or worldwide. *S. pneumoniae*^Spain23F^ ST81 was one of the first pandemic penicillin-resistant clones identified^5^. Analysis of the complete genome of *S. pneumoniae* ATCC 700669, a member of the serotype 23F ST81 lineage, indicated that integrative and conjugative elements, which provide a large gene pool including antibiotic resistance, facilitated the rapid adaptation of this clone to new selective pressure and was responsible for the clone’s international success^6^. Clonal success of *S. pneumoniae* was not solely due to antibiotic resistance, as evidenced by the dissemination of non-antibiotic resistant clones. The serotype 14 ST 124 clone represented one of the most successful penicillin-susceptible clones in Scandinavia, the United Kindom, the Netherlands, and Australia; the genetic basis for this widespread dissemination remains unkown^7^. It is thought that genetic factors other than antibiotic resistance also contribute to clonal success. For example, the successful global expansion of the Spain^9V^-3 clone (ST156) was attributed to the presence of *rlrA* pilus islet, which promotes colonization as well as virulence of *S. pneumoniae*^8^. Pneumococcal serine-rich repeat protein (PsrP), a pathogenicity island encoded adhesin, was positively correlated with the ability of *S. pneumoniae* to cause invasive disease^9^.

In this study, we constructed a microarray based on the genome of an endemic, multi-resistant serotype 14 ST 46 clone^2^. Genomic variation among a clinical strain (NTUH-P15) of serotype 14 ST 46 and 3 non-clonal-expansion strains of pneumonia were compared by DNA microarray hybridization to obtain insights into the mechanism of expansion of the serotype 14 ST 46 clone in Taiwan.

## Results

### DNA microarray hybridization

**Figure 1:**
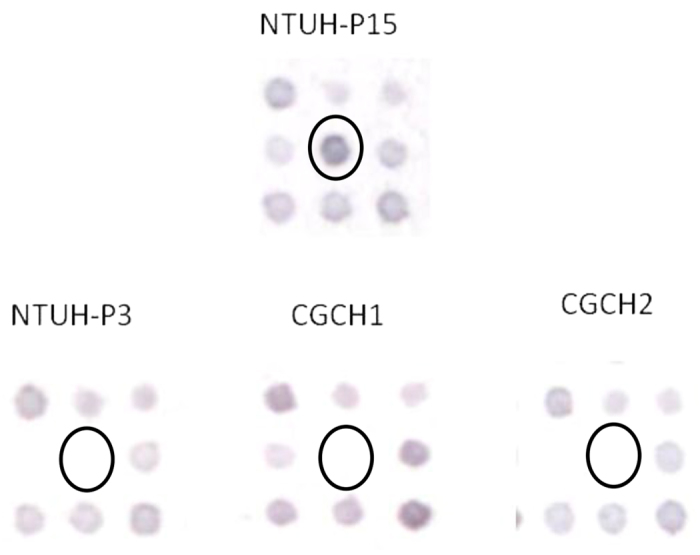
DNA hybridization and colorimetric detection of NTUH-P15 and 3 non-clonal expansion strains on microarray analysis. Each spot represents one clone. The spot showing significant increases in hybridization signals in the NTUH-P15 strain compared with the other three strains are circled. Spots showing no significant increases in hybridization signals in the NTUH-P15 strain compared with the other three strains are not circled.

Comparison of microarray results for NTUH-P15 and 3 control strains (NTUH-P3, CGCH1 and CGCH2) revealed 7 “spots” (plasmid clones) that had significantly higher hybridization signals (defined as >10-fold differences) in the NTUH-P15 strain. The inserts of these 7 plasmid clones were sequenced and subjected to sequence similarity (BLAST) searches. Genes contained in each clone are shown in Table 1. Blast searches of clone 1 identified that clone 1 contained genes with similarity to the adjacent loci SPP_0074 and SPP_0075 of *S. pneumoniae* P1031 (Table 1); the products of these genes exhibit similarity to a host specificity protein and PblB, respectively (Fig. 1). From literature review, PblB of *Streptococcus mitis*, a phage-encoded virulence factor, was implicated in infective endocarditis^10^. Therefore, we chose plasmid clone 1 for further study.

### DNA and amino acid sequence analysis of clone 1

**Figure 2:**
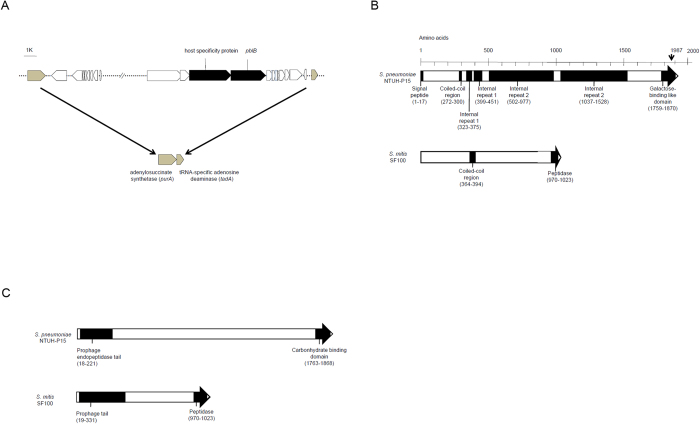
Schematic representation of PblB of *Streptococcus pneumoniae*. **A**. The host specificity protein gene and *pblB* gene (black squares) residing within a 33-kb temperate bacteriophage located in an insertion site between the *purA* (adenylosuccinate synthetase) and *tadA* (tRNA-specific adenosine deaminase) (gray squares) genes in *Streptococcus pneumoniae* P1031. **B**. Analysis by using the SMART program (http://smart.embl-heidelberg.de/). PblB of *Streptococcus pneumoniae* NTUH-P15 is composed of a signal peptide, a coiled-coil region, four internal repeats, and a galactose-binding domain-like region. PblB of *Streptococcus mitis* SF100 is composed of a coiled-coil region and a peptidase domain. **C**. Analysis by using BLAST. PblB of *Streptococcus pneumoniae* NTUH-P15 is composed of a prophage endopeptidase tail and a carbonhydrate binding domain. PblB of *Streptococcus mitis* SF100 is composed of a prophage tail and a peptidase domain.

Analysis of the complete genome of the P1031 strain suggested that the host specific protein gene and *pblB* gene reside within a 33-kb temperate bacteriophage located in an insertion site between the *purA* (adenylosuccinate synthetase) and *tadA* (tRNA-specific adenosine deaminase) genes (Fig. 2A). We cloned and sequenced the corresponding chromosomal region (i.e., flanking the host specific protein gene and *pblB* gene) from NTUH-P15. A total of 7.1 kb, including 0.7 kb upstream and 0.5 kb downstream, was sequenced. Analysis of sequence suggested that the host specific protein gene and *pblB* gene of NTUH-P15 would be transcribed together, in contrast to the separate transcription predicted for the loci in *S. pneumoniae* P1031. PblB of NTUH-P15 (GenBank accession number AB679266) is predicted to encode a 213-kDa protein with a pI of 8.98. By Pairwise Sequence Alignment (www.ebi.ac.uk/Tools/psa/), the predicted PblB of NTUH-P15 shared 24.3% sequence identity and 40.3% similarity with PblB in *S. mitis*. On the basis of its amino acid sequence, PblB is predicted to form a signal peptide, a coiled-coil region, 4 internal repeats, and a galactose-binding domain-like region located at the carboxy terminus by the SMART program (http://smart.embl-heidelberg.de/) (Fig. 2B). By BLAST analysis, PblB is predicted to form a prophage endopeptidase tail and a carbonhydrate binding domain (Fig. 2C).

### Prevalence of *pblB* gene among *S. pneumoniae* strains

Primers pblB F4 and pblB R4 (Table 2) were used to detect the presence of the *pblB*-positive strains. The three control strains were confirmed to be *pblB*-negative strains. Among 77 invasive pneumococcal isolates causing pneumonia, all strains belonging to the largest clone (serotype 14 ST 46) and the second largest clone (serotype 6B ST 76) harbored *pblB*. The prevalence of *pblB* gene is significantly higher in serotype 14 ST 46 strains and serotype 6B ST76 strains compared to that in strains not belonging to either of these genotypes (25/25 (100%), vs. 16/52 (30.8%), respectively (*P* < 0.001) (Table 3). PblB of two strains of serotype 14 ST46 clone and serotype 6B ST76 clone were randomly selected to analyze their sequences. The predicted PblB of serotype 14 ST46-1, serotype 14 ST46-2, serotype 6B ST76-1 and serotype 6B ST76-2 shared 95.9%. 96.1%, 71% and 71% sequence similarity with PblB in NTUH-P15. Serotype 14 ST46-1 and ST46-2 have the same sequences of galactose-binding domain-like region as NTUH-P15 does (amino acid 1759–1870) (Supplementary Figure 1). Serotype 6B ST76-1 and ST76-2 have sequence variation in the area, but both strains also have galactose-binding domain-like region (amino acid 1449–1516) predicted by the SMART program.

### PblB contribute to adhesion of *S. pneumoniae* to platelets, A549 and HEp2 cells

**Figure 3:**
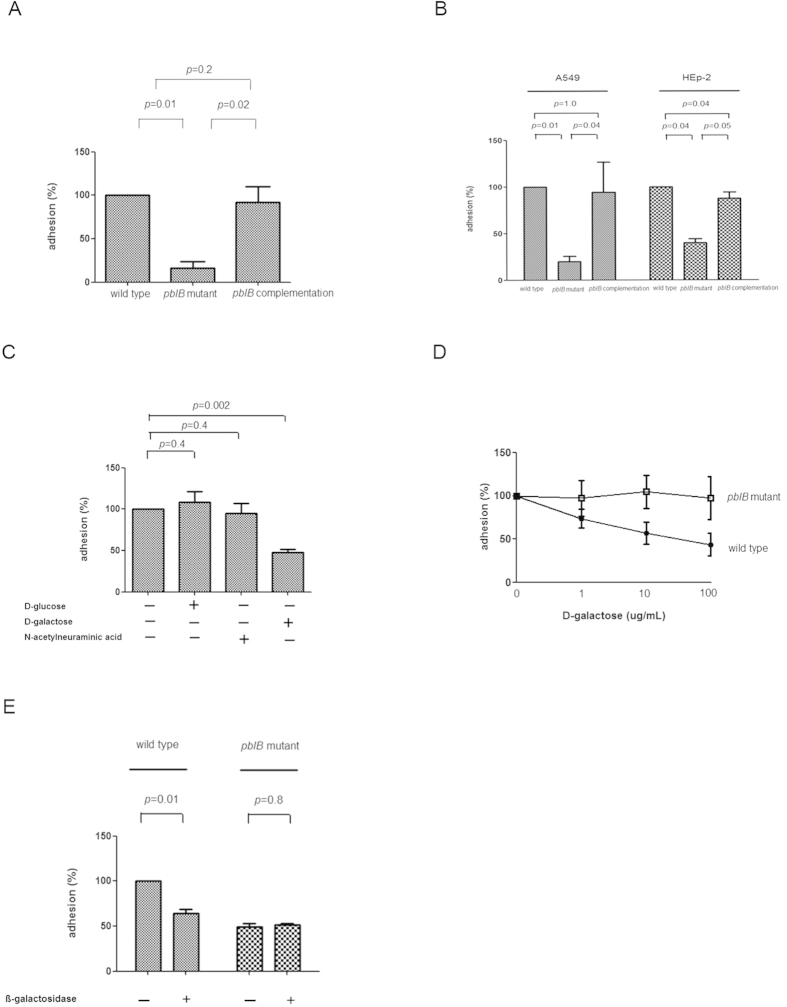
Adherence of wild-type NTUH-P15, *pblB* mutant, and *pblB* complementation strains to platelets/A549/HEp-2 cells *in vitro*. **A**. The *pblB* mutant decreased adherence to platelets compared to the wild-type strain. Complementation with *pblB* restored the adherence. **B**. The *pblB* mutant decreased adherence to A549 and HEp-2 cells compared to the wild-type strain. Complementation with *pblB* restored the adherence. **C**. Preincubation of wild-type NTUH-P15 with D-galactose decreased adherence to A549 cell. In contrast, preincubation of wild-type NTUH-P15 with D-glucoase and N-acetylneuraminic acid did not decreased adherence to A549 cell. **D**. Adherence of NTUH-P15 wild-type to A549 cells was inhibited in a dose-dependent manner by pretreatment of bacteria with D-galactose, but not in the isogenic *pblB* mutant. **E**. Adherence of NTUH-P15 wild-type to A549 cells pre-treated with ß-galactosidase decreased compared to that without ß-galactosidase, but not in the isogenic *pblB* mutant. Experiments were performed in triplicate. The standard error of the mean (SEM) are indicated as bar graph.

Growth curves of the wild-type, mutant and complementation strains were comparable (data not shown). Since PblB is a platelet adhesin in *S. mitis*^10^, we evaluated whether PblB of NTUH-P15 mediates adhesion to platelets. We constructed an isogenic *pblB* insertion mutant in the NTUH-P15 strains. The *pblB* mutant decreased adherence to platelets (*P* = 0.01) compared to the wild-type strain (Fig. 3A). When the NTUH-P15 *pblB* mutant strain was complemented with the *pblB* gene, the adherence of the wild-type strain to platelets (*P* = 0.2) were not significantly different from the adherence of the complementation strain (Fig. 3A). Subsequently, we measured the impact of *pblB* on pneumococcal adherence to respiratory epithelial cell. The *pblB* mutant decreased adherence to lung epithelial cell (A549 cells) (*P* = 0.01) and laryngeal cell (HEp-2) (*P* = 0.04) compared to the wild-type strain (Fig. 3B). When the NTUH-P15 *pblB* mutant strain was complemented with the *pblB* gene, the adherence of the wild-type strain to A549 cells and HEp-2 were restored (Fig. 3B). Introduction of *pblB* into the *pblB*-negative NTUH-P3 strain did not increase adherence to A549 cells as compared to that of *pblB*-negative NTUH-P3 strain (*P* = 0.8) (data not shown)

### PblB mediate adhesion though binding to galactose-containing glycoconjugates on host cells

PblB was predicted to contain a galactose-binding domain-like region at the carboxy terminus. We assumed that PblB adhere to respiratory epithelial cell through binding to galactose. Preincubation of NTUH-P15 wild-type with D-galactose (100 ug/mL) significantly decrease adherence to A549 cell, compared with those without preincubation with D-galactose (*P* = 0.002) (Fig. 3C). In contrast, preincubation of NTUH-P15 wild-type with D-glucose (100 ug/mL) (*P* = 0.4) and N-acetylneuraminic acid (100 ug/mL) (*P* = 0.4) did not decrease adherence to A549 cell (Fig. 3C). Moreover, adherence of NTUH-P15 wild-type was inhibited in a dose-dependent manner by pretreatment of bacteria with D-galactose (Fig. 3D). Treating A549 cells with ß-galactosidase resulted in an approximately 40% decrease in the adhesion of NTUH-P15 wild-type (*P* = 0.01) (Fig. 3E).

### PblB is required for nasopharyngeal and lung colonization

**Figure 4:**
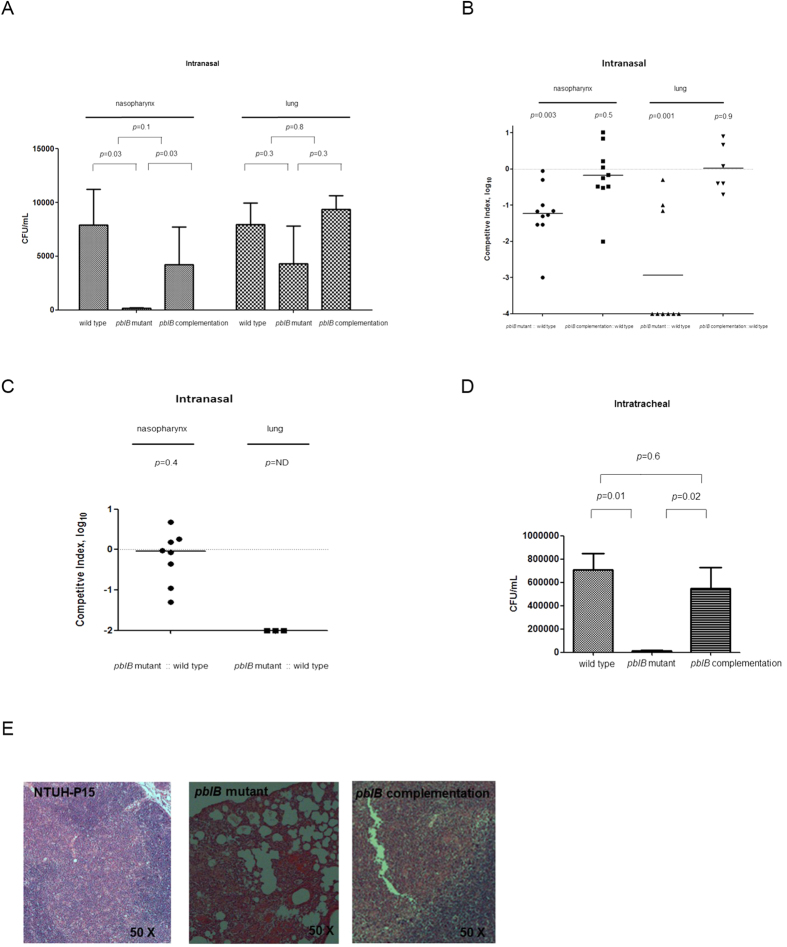
Mouse challenge. **A**. Intranasal challenge of 3-week-old female BALB/c mice (n = 4–6 per group in the separate model) with 5 × 10^7^ colony-forming units (CFUs) of wild-type NTUH-p15, *pblB* mutant, and *pblB* complementation strains. Seven days later, the *pblB* mutant had a significantly lower bacterial titers in the nasopharynx than that of mice infected with strain NTUH-P15, but not in the lungs. Complementation with *pblB* restored bacterial titers in the nasopgaryngx. Intranasal challenge of 3-weeks-old female BALB/c mice (n = 6–10 per group in the competition model) with equal inocula of bacterial strains. Each symbol represents the competitive index (CI) value for an individual animal. CI was calculated as described in Methods. Briefly, CI indicates the log_10_ normalized ratio. Horizontal bars indicate the median. **B**. Seven days later, NTUH-P15 wild-type strain significantly out-competed the NTUH-P15 *pblB* mutant in the nasopharynx and lung. A CI below 0 indicates a competitive disadvantage of the mutant in relation to the wild-type strain. When the NTUH-P15 *pblB* mutant strain was complemented with the *pblB* gene, NTUH-P15 wild-type strain did not out-compete the complementation strain in the nasopharynx and lung. **C**. Galactose preincubation before intranasal inoculation. Seven days later, NTUH-P15 wild-type strain did not significantly out-compete the NTUH-P15 *pblB* mutant in the nasopharynx, but out-competed the NTUH-P15 *pblB* mutant in the lung. ND: not done (can’t be calculated). Intratracheal challenge of 3-week-old female BALB/c mice (n = 4–6 per group in the separate model) with 5 × 10^7^ colony-forming units (CFUs) of wild-type NTUH-p15, *pblB* mutant, and *pblB* complementation strains. **D**. Twenty-four hours after inoculation, the *pblB* mutant had a lower bacterial titers in the lung than that of mice infected with strain NTUH-P15. Complementation with *pblB* restored bacterial titers in the lung. **E**. Twenty-four hours after inoculation, strain NTUH-P15 had extensive and increased infiltration of inflammatory cells infiltration in the lung than that of mice infected with the *pblB* mutant. Complementation with *pblB* restored the severity of pneumonia. H&E-stained tissue samples.

Intranasal challenge of mice infected with the *pblB* mutant had a significantly lower bacterial titers in the nasopharynx than that of mice infected with strain NTUH-P15 (*P* = 0.03), but not in the lungs (Fig. 4A). When the NTUH-P15 *pblB* mutant strain was complemented with the *pblB* gene, bacterial titers in the nasopgaryngx could be restored (Fig. 4A). To reduce the variance in the experimental setup caused by variation between individual mice and inoculation efficacy, we performed competitive experiment. Seven days after intranasal inoculation, the NTUH-P15 wild-type strain significantly out-competed the NTUH-P15 *pblB* mutant in the nasopharynx (*P* = 0.003) and lung (*P* = 0.001) (Fig. 4B). When the NTUH-P15 *pblB* mutant strain was complemented with the *pblB* gene, NTUH-P15 wild-type strain did not out-compete the complementation strain in the nasopharynx (*P* = 0.5) and lung (*P* = 0.9) (Fig. 4B). Galactose was preincubated with strain NTUH-P15 and the NTUH-P15 *pblB* mutant strain before mixing together and intranasal inoculation. Seven days after intranasal inoculation, the NTUH-P15 wild type strain did not significantly out-compete the NTUH-P15 *pblB* mutant in the nasopharynx (*P* = 0.4) (Fig. 4C). In the lung, the NTUH-P15 wild type strain still out-competed the NTUH-P15 *pblB* mutant (*P* value can’t be calculated) (Fig. 4C). Comparing to strain NTUH-P15 competing with the NTUH-P15 *pblB* mutant strain without galactose pre-incubation, a significantly lower percentage of mice was colonizing with pneumococci in the lung when strain NTUH-P15 competing with the NTUH-P15 *pblB* mutant strain with galactose pre-incubation (37.5% (3/8, Fig. 4C) vs 90% (9/10, Fig. 4B); *P* = 0.04). Intratracheal challenge of mice infected with the *pblB* mutant had a significantly lower bacterial titers in the lung than that of mice infected with strain NTUH-P15 at 24 hour post-challenge (*P* = 0.03) (Fig. 4D), but not at 48 hour (data not shown). When the NTUH-P15 *pblB* mutant strain was complemented with the *pblB* gene, bacterial titers in the lung could be restored at 24 hour post-challenge (Fig. 4D). Microscopic evaluation show extensive and increased infiltration of inflammatory cells in the lung caused by strain NTUH-P15 at 24 hour post-challenge compared to that caused by the NTUH-P15 *pblB* mutant strain (Fig. 4E). *PblB* complementation strain restored the severity of pneumonia (Fig. 4E). The survival rates between wild type and mutant strains were not significantly different.

## Discussion

Our findings identified that *pblB*, a phage-encoded protein in a successful invasive pneumococcal clone, contributes to pneumococcal adhesion through binding to galactose-containing glycoconjugates on lung epithelial cell.

Phages, the most abundant entities in nature, may constitute 20% of bacterial genomes and play a central role in the shaping of natural populations of bacteria^11^. It is known that temperate bacteriophages can confer higher fitness on a host by coding for genes that enhance host virulence or influence host physiology. For example, the Shiga toxin produced by *Escherichia coli* O157:H7, the β-toxin produced by *Corynebacterium diphtheria*, the endotoxin produced by *Clostridium botulinum*, and the cholera toxin produced by *Vibrio cholerae* each contribute to bacterial pathogenesis^12^. Prophage elements in *E. coli* K-12 help the bacterium increase growth and biofilm formation, and enhance the bacterium’s response to stress and to antibiotics^13^. The presence of temperate bacteriophages in pneumococcal isolates is quite high. Up to 76% of clinical pneumococcal isolates harbor temperate bacteriophages^14^. Nevertheless, the function of many of the genes that reside in the genome of pneumococcal temperate bacteriophage were not clear^15^. MM1, isolated from a Portuguese clinical pneumococcal strain, is the only phage that has been demonstrated to improve adherence to pharyngeal cells, an activity that may confer an advantage in colonization^16^.

PblB proteins were initially identified as surface proteins that are involved in the platelet binding activity of *S. mitis* causing infective endocarditis^10^. The *S. mitis pblB* gene resides within the temperate bacteriophage SM1, a member of the *Siphoviridae* family^11^. PblB of *S. mitis* functions in adhesion by interacting with α 2-8-linked sialic acid residues on platelet membrane gangliosides^17^, although the protein also shows homology to phage tail fiber protein and is important for tail morphogenesis^10^. Studies have shown that induction of the phage lytic cycle results in permeabilization of *S. mitis* and associated release of PblB, which binds to other viable bacteria^18^. Romero *et al*. divided 10 *S. pneumoniae* temperate bacteriophages into three groups^15^. *PblB*-like genes were identified among group 1 and 2 phages. The PblB protein of *S. pneumoniae* NTUH-P15 shares only 19% identity with PblB in *S. mitis*. In our study, we evaluate whether PblB contributes to adhesion to respiratory epithelial cell. The result indicated that PblB is involved in the adherence to human epithelial cell, and galactose-residue on the surface of lung epithelial cell is the likely target receptor for pneumococcal PblB. Most human cell surfaces are glycosylated with a number of different sugar residues which act as receptors for a wide variety of bacterial adhesion, like *Escherichia coli* P fimbriae as well as Shiga toxins of *Shigella dysenteriae* and *Pseudomonas aeruginosa* lectin^19^,^20^,^21^. In pneumococci, there were studies provided evidence that pneumococci has an adhesive interactions with host cell glycoconjugates^22^,^23^, but which bacterial genetic factor responsive for the mechanism remain not clear. In this study, we observed that PblB promote pneumococcal adherence by mediating the galactose-specific adhesion activity to respiratory epithelial cell. A putative signal peptide was found in the N terminus of PblB, indicating that the protein is exported from the pneumococcus. Furthermore, a prophage endopeptidase tail was also predicted at amino acid 18–221 position. Tails of bacteriophages usually specifically recognize the target bacteria and serve adhesion properties^24^. We hypothesized that PblB might use its N-terminal prophage tail domain to associate with pneumococcal surface and its C-terminal galactose binding domain to adhere human cell. Adding pblB into NTUH-P3 which did not harbor the temperate bacteriophage did not increase adherence to A549 cells. This result indicates that PblB mediating adhesion may need other phage component, not only PblB. Further work, as purifying the galactose binding domain and doing biochemical tests, is needed to prove this domain is functional.

Colonization is a prerequisite for pneumococcal disease. In a murine model of colonization, mutation of *pblB* impaired pneumococcal colonization. The *pblB* gene was detected not only in pneumococcal isolates of serotype 14 ST 46, but also in the second most prevalent pneumococcal clone, that of serotype 6B ST 76. PblB provides a competitive advantage for the pathogen’s persistence in the respiratory tract to cause diseases. In conclusion, PblB is responsible for a clonal property characterized by enhancing pneumococcal colonization and pneumonia. The study expands our understanding of the impact of phages on the evolution of the *S. pneumoniae* genome.

## Methods

### *S. pneumoniae* strains and culture conditions

The institutional review board of the Chang Gung Memorial Hospital approved this study, and the methods were carried out in accordance with the approved guidelines. Samples and data for children with invasive pneumococcal infection were collected after written informed consents were obtained from every subject’s parents or legal guardians. A total of 77 *S. pneumoniae* strains of pneumonia from culture of blood or pleural fluid were obtained from patients <18 years of age who had been diagnosed with pneumococcal pneumonia at Chang Gung Children’s Hospital (CGCH) between 2001 and 2005. All pneumococcal isolates were grown at 35 °C in Todd–Hewitt broth supplemented with 0.5% yeast extract (THY) in static culture in the presence of 5% CO_2_. The serotypes of isolates were determined by using the capsular swelling method (Quellung reaction). All antisera were obtained from the Statens Serum Institut (Copenhagen, Denmark). Multilocus sequencing type (MLST) was determined as described previously^25^.

### Microarray construction

NTUH-P15 is a clinical isolate of serotype 14 ST 46 that was recovered from the blood of a child with necrotizing pneumococcal pneumonia and empyema. Genomic DNA of NTUH-P15 was prepared as described previously^3^. Aliquots of 20 μg of genomic DNA were partially digested with *Sau*3AI and separated by agarose gel electrophoresis. DNA fragments of 1 to 3 kb were extracted from agarose gel and then ligated to the *Bam*HI site of plasmid pUC19. A total of 2,880 plasmid clones were amplified by PCR with primers designed using vector flanking sequences (Table 2), and all of the resulting amplicons were used for the microarray. Amplicons were spotted onto a nylon membrane (Roche) by a computer-controlled XYZ translation system (PM500; Newport)^26^. The coverage rate of the genomic library was ~85%, according to the formula *N* = ln (1-*P*)/ln(1-*f* )^27^. To test the redundancy of the library, 12 of the 2,880 plasmid clones were randomly selected for sequencing. These 12 clones revealed 11 distinct sequences (i.e., 8.3% redundancy).

### Probe preparation and hybridization

We chose three strains, NTUH-P3 (serotype 14 ST 329), CGCH1 (serotype 19F ST 236), and CGCH2 (serotype 23F ST 83) as controls for comparison to serotype 14 ST 46 clones. These three strains were isolated from the blood of children with pneumococcal lobar pneumonia. In our previous studies, the prevalence rates of these three clones among strains of pneumonia were below 5%^2^,^4^. Genomic DNA from NTUH-P15 and the 3 control strains (NTUH-P3, CGCH1 and CGCH2) were extracted and were labeled with biotin-11-dUTP (Perkin Elmer) by a randomly primed polymerization reaction^28^. The microarray membrane was pre-hybridized in 2 mL of hybridization buffer for 4 h at 65 °C before hybridizing with labeled probe for 16 h at 68 °C. The membrane was washed twice with 2× standard saline citrate (SSC) containing 0.1% SDS for 5 min (per wash) at room temperature and then was washed 3 times with 0.1× SSC containing 0.1% SDS for 15 min (per wash) at 65 °C. Colorimetric detection and image analysis then were performed as described elsewhere^29^.

### Construction of *pblB* insertion mutants

First, the kanamycin resistance-encoding cassette on the EZ-Tn5 MOD (Epicentre®) vector was replaced by a chloramphenicol (Cm) resistance-encoding cassette to generate plasmid EZ-Tn5Cm. An approximately 0.6-kb DNA fragment (containing the partial coding sequences of the *pblB* gene) was amplified using primers pblB F4 and pblB R4 and cloned into the pGEM-T Easy vector (Promega) to generate plasmid pGEM-T Easy-pblB. This plasmid was subjected to *in vitro* mutatgenesis using EZ-Tn5Cm, and the resulting products were transformed into *E. coli* with selection for Cm resistance. To generate chromosomal *pblB* insertion mutants, the pGEM-T Easy-pblB::Tn5Cm plasmid was transformed into wild-type *S. pneumoniae* NTUH-P15 using CSP-1^3^. Insertion mutants were selected using 4 μg/mL of Cm. Insertion mutants were confirmed by PCR with multiple primer pairs and sequence determination. The primers used are listed in Table 2.

### Construction of the *pblB* complementation strains and introduction of *pblB* into a *pblB*-negative pneumococcal strain

Complementation of *pblB* in NTUH-P15 was achieved by inserting a copy of the *pblB* gene of NTUH-P15 into the noncoding region between the SP0484 and SP0489 genes on the chromosome of the NTUH-P15 *pblB* mutant^3^. To do this, an approximately 2-kb fragment between the SP0484 and SP0489 genes was amplified by primers 484F and 489R, then cloned into the pGEM-T Easy vector to yield pGEM-T Easy::SP0484–0489. *PblB*, together with its ribosome-binding site and promoter predicted by Promotor 2.0 Prediction Server (www.cbs.dtu.dk/Services/Promotor), was amplified by PCR using primers 0073F and 0076R; the resulting fragment was cloned into the pGEM-T Easy vector. The *spec* marker (encoding spectinomycin (Spec) resistance) was inserted into the *Sac*II site of pGEM-T Easy::*pblB*, and flanking primers (T7 and PblB R5) were used to amplify *spec* and the complete *pblB* gene as a single fragment. The complete *pblB* gene with *spec* fragment was ligated with the inverse PCR product of pGEM-T Easy::SP0484-048 amplified by using primers 486F (iPCR) and 487R (iPCR), and the resulting plasmid then was used to transform NTUH-P15 *pblB* insertion mutants using CSP-1. Complementation strains of NTUH-P15 were selected using 500 μg/mL of Spec and 4 μg/mL of Cm; chromosomal gene constructs were confirmed by PCR and DNA sequencing. The control strain NTUH-P3 was a *pblB*-negative serotype 14 strain. To create a *pblB*-positive NTUH-P3, NTUH-P3 was transformed with the pGEM-T Easy::SP0484-spec-*pblB*^+^-0489 plasmid. The double recombination event was selected using 500 ug/ml of Spec, and *pblB* presence was confirmed by PCR.

### Adherence assay

The A549 (ATCC CCl-185; type II pneumocytes) and HEp-2 (ATCC CCL23; human laryngx carcinoma) cells were grown in Dulbecco’s modified Eagle’s medium (DMEM) with 10% fetal bovine serum and seeded in the wells of a 24-well plate and cultivated to confluent cell layers with approximately 2 × 10^5^ cells per well^30^. Before use, the monolayers were washed three times with PBS. Bacteria were grown to the early log phase, washed, resuspended, and applied to the monolayers at a multiplicity of infection of 1:100. The plates were centrifuged at 800 × *g* for 10 min, and then were incubated for 1 h at 37 °C with 5% CO2 and washed with PBS for three times to remove nonadherent bacteria. The number of adhering bacteria was determined by lysis of the monolayer with 0.1% Triton X-100 and plating of the lysate. Adherence results were expressed as a percentage of wild type NTUH-P15 adhesion to A549 cells^30^. For galactose, glucose, N-acetylneuraminic acid inhibition assay^31^, 2 × 10^7^ cfu/ml of log phase *S. pneumoniae* were preincubated with 100 ug/ml of D-galactose, D-glucose and N-acetylneuraminic acid, respectively, for 10 min at room temperature, then were added on A549 cell. For dose-dependent galactose inhibition assay, bacteria were preincubated with two additional doses of D-galactose (1 and 10 ug/ml). For the deglycosylation of A549 cell^31^, the A549 cell were incubated with DMEM media containing 160 nM purified ß-galactosidase (Sigma) from *S. pneumoniae* at 37 °C for 4 hr in 5% CO2.

### Platelet adhesion assay

Platelet adhesion was measured as described previously^10^,^17^,^32^. A total of 8.5 volumes of blood were added to 1.5 volume of acid-citrate-dextrose (ACD, 25 mM citric acid, 75 mM sodium citrate, 135 mM D-glucose). Whole blood was subsequently centrifuged at 150 × g for 10 min. The top layer consisting of platelet rich plasma (PRP) was collected. Following preparation of PRP, the pH of the platelets was adjusted to 6.5 using ACD. Prostaglandin E1 (1 μM) was added to the platelets. PRP was centrifuged at 650 × g for 10 min. The supernatant was carefully removed and discarded. One ml of tyrode’s solution (acidic) was layered on top of the platelet pellet and then counting cell number. Fixed 5 × 10^7^ human platelets were immobilized in poly-L-lysine coated 22-mm-diameter tissue culture wells. After 30 min at 37 °C, the unbound platelets were removed by aspiration. To reduce non-specific adherence, the wells were then treated with a 1% casein solution in DPBS for 1 hr at room temperature. After the blocking solution was removed by aspiration, the wells were inoculated with 5 × 10^9^ CFU of bacteria in 1 ml of DPBS and incubated at 37 °C for 2 hr. Then, the medium was removed and the platelets were washed for 3 times with PBS. To lyse the cells and detach the adhered bacteria, add 1 ml ddH_2_O to each well containing the cells, and then incubate 10 min at room temperature. Gently homogenize the suspensions by repeated up-and-down pipetting. Prepare serial 10-fold dilutions of the suspensions of adhered bacteria and inoculum using THY broth and plate 100 μl from 3 dilutions (usually the 1:10, 1:100, 1:1000 dilutions) on blood agar and incubate overnight at 37 °C.

### Mouse challenge

Animal studies were performed using 3-week-old female BALB/c mice. For intranasal challenge, mice were anesthetized with 20 mg/kg ketamine and inoculated intranasally with 5 × 10^7^ colony-forming units (CFU)s of bacteria. At 7 days post-challenge, the animals were sacrificed. Nasal lavages and lung homogenates were collected as previously described and subjected to serial dilution and plated to blood agar to determine the number of viable pneumococci^33^. For the competition model^33^, bacteria were combined at 1:1 ratios of CFUs. Twenty microliters (5 × 10^7^ CFUs) were used for intranasal challenge. For competitions between wild type and the *pblB* mutant, recovered organisms were distinguished by replica plating to blood agar containing 4 μg/mL Cm. For competitions between wild type and the complementation strains, using the same method, the numbers of complementation strains were determined by replica plating onto blood agar containing 4 μg/mL of Cm and 500 μg/mL of Spec. A competitive index (CI) was calculated as previously described^8^,^33^. For intratracheal challenge, mice were inoculated intratracheally with 5 × 10^7^ CFUs of bacteria^3^. To test the effect of galactose on pneumococcal colonization in competition model, both bacteria were preincubated with 100 ug/ml of D-galactose for 10 min at room temperature before mixed together at 1:1 ratio of CFUs. Animal experiments were carried out in strict accordance with the Guide for the Care and Use of Laboratory Animals, Institute of Laboratory Animal Resources Commission on Life Sciences National Research Council, USA, and all efforts were made to minimize suffering. All animal procedures were approved by the Institutional Animal Care and Use Committee (IACUC) of Chang Gung University for the use of laboratory animals (Permit Number: CGU12-080).

### Statistical analysis

The chi-square test was used to compare the prevalence of *pblB* among clinical strains. To test significance between groups, the Mann-Whitney test was used for continuous variables. To determine if the mouse CI were significantly less than 1, CI values were log-transformed and analyzed with a one-sample Student’s *t* test. A *P*-value < 0.05 was considered statistically significant. All analyses used the SPSS statistical package (SPSS Inc., Chicago, IL, USA).

## Additional Information

**How to cite this article**: Hsieh, Y.-C. *et al*. Identification of PblB mediating galactose-specific adhesion in a successful *Streptococcus pneumoniae* clone. *Sci. Rep*. **5**, 12265; doi: 10.1038/srep12265 (2015).

## Supplementary Material

Supplementary Information

## Figures and Tables

**Table 1:** Identification of 7 clones that had significant increases in hybridization signals of NTUH-P15 compared with 3 Control strains.

Clone no.	gene	Predicted protein	GenBank accession no.
1	SPP 0074	Host specificity protein	CP000920.1
	SPP 0075	PblB	
2	SPCG 0649	HesA/MoeN/ThiF family protein	CP001033.1
	SPCG 0650	ABC transporter ATP-binding protein	
3	SPP 1758	Conserved hypothetical protein	CP000920.1
	SPP1759	Conserved hypothetical protein	
4	SPT 0236	phage protein	CP000921.1
5	SP 0167	hypothetical protein	AE005672.3
	SP 0168	putative macrolide efflux protein	
	SP 0169	lactose phosphotransferase system repressor	
	SP 0170	hypothetical protein	
	SP 0171	ROK family protein	
	SP 0172	hypothetical protein	
	SP 0173	DNA mismatch repair protein HexB	
6	SP70585 1097	protein tyrosine phosphatase, putative	CP000918.1
	SP70585 1098	ABC transporter, ATP-binding protein	
	SP70585 1099	ABC-type transport system, authentic frameshift	
	SP70585 1101	ABC transporter permease protein	
7	SPJ 0069	membrane protein, putative	CP000919.1
	SPJ 0070	hypothetical protein	
	SPJ 0071	hypothetical protein	
	SPJ 0072	hypothetical protein	

**Table 2:** DNA primers used in this study.

Primer	Sequence	Description
Xg-F	5′-TGC AAGGCG ATT AAG TTGGGT A-3′	pUC 19 primer
Yg-R	5′-CAGGAA ACAGCT ATGACC-3′	pUC 19 primer
pblB F4	5′-GGT CAG CGA CAG GAA GCC CTA-3′	*pblB* mutant and *pblB*-positive prevanlance
pblB R4	5′-CGC AAT TCC ATT TTC AGT ATT-3′	*pblB* mutant and *pblB*-positive prevanlance
pblB F1	5′-AAT CAG CGC TTT AAT AGC-3′	confirm mutant
sp484 F	5′-AGC CTG ACC TTG CGC TAT ATT CC-3′	*pblB* complementation
sp489 R	5′-GAA TAG TCA AAT AAT CTG GTA AGT CTC C-3′	*pblB* complementation
486F (iPCR)	5′-GTCACCGCCGGTCGGGAA-3′	*pblB* complementation
487R (iPCR)	5′-GCCTACAGCTTGTCGCCTAG-3′	*pblB* complementation
0073F	5′-AGG TTC TGC TCT TGC CTT TCC-3′	*pblB* complementation
0076R	5′-CCG ACC TGA TTC CAC CAA ACA-3′	*pblB* complementation
T7	5′-TAA TAC GAC TCA CTA TAG GG-3′	*pblB* complementation
pblB-R5	5′-CTA ATA TCG TAG TCG TGA CCG-3′	*pblB* complementation confirm mutant
484 (−200F)	5′-CGA GTA TGG GGT TGG ACT TTA TGG AGA GAG-3′	confirm complementation
488 (+100R)	5′-TGC CAA AGC CAG ATT TCC CA-3′	confirm complementation

**Table 3:** Prevalence of *pblB* gene among clinical *Streptococcus pneumoniae* strains.

Strains of pneumonia (n = 77)	Clinical characteristics of strains^a^		PblB
	Underlying disease	Source/ Disease	
Serotype 14			
ST46 (n = 14)	1	B(7), P(4), B+P(3) / non-CP(4), CP(10)	100% (14)
ST13 (n = 5)	0	B(3), P(1), B+P(1) / non-CP(2), CP(3)	40% (2)
ST876 (n = 7)	1	B(7) / non-CP(5), CP(2)	14.3% (1)
Serotype 6B			
ST76 (n = 11)	2	B(8), P(1), B+P(2) / non-CP(8), CP(3)	100% (11)
ST95 (n = 8)	2	B(7), B+P(1) / non-CP(6), CP(2)	75% (6)
Serotype 3			
ST180 (n = 6)	1	B(4), P(1), B+P(1) / non-CP(2), CP(4)	16.7% (1)
Serotype 23F			
ST83 (n = 2)	1	B(2) / non-CP(2), CP(0)	50% (1)
ST81 (n = 3)	1	B(2), P(1) / non-CP(2), CP(1)	0% (0)
ST 242 (n = 3)	1	B(3) / non-CP(3), CP(0)	0% (0)
Serotype 19F			
ST3182 (n = 2)	0	B(2) / non-CP(2), CP(0)	50% (1)
Others (n = 16)	3	B(6), P(7), B+P(3) / non-CP(6), CP(10)	25% (4)

^a^Abbreviations: B, blood; P, pleural; non-CP, non-complicated pneumonia; CP, complicated pneumonia
