# Supplementary material for: Identification of PblB mediating galactose-specific adhesion in a successful Streptococcus pneumoniae clone
Source: Sci Rep. 2015 Jul 21;5:12265. doi: 10.1038/srep12265 (PMC4508584; doi:10.1038/srep12265)
Supplement: Supplementary Information [file srep12265-s1.pdf]

Identification of PblB mediating galactose-specific adhesion in a successful *Streptococcus pneumoniae* clone

Yu-Chia Hsieh[1],Tzu-Lung Lin[2], Che-Ming Lin[1], Jin-Town Wang[2,3,*]

[1]*Department of Pediatrics, Chang Gung Children's Hospital, Chang Gung Memorial Hospital, Chang Gung University, College of Medicine, Taoyuan, Taiwan;*[2]*Departments of Microbiology, National Taiwan University College of Medicine, Taipei, Taiwan;*[3]*Departments of Internal Medicine, National Taiwan University Hospital, Taipei, Taiwan*

*Corresponding author: Professor Jin-Town Wang

Department of Microbiology, National Taiwan University College of Medicine

No 1, Sec 1, Jen-Ai Rd, Taipei, Taiwan 10051

Tel: +886-2-2312-3456, ext.88292; Fax: +886-2-2394-8718

E-mail: wangjt@ntu.edu.tw

Key Words: *Streptococcus pneumoniae*; microarray; phage; adhesin; galactose

Running Title: *pblB* of *Streptococcus pneumoniae*

CLUSTAL 2.1 multiple sequence alignment

```
NTUH-P15        ---------------MAWLFLAWYKTPKRALGDFSFNSDIQDRRTFNTTETETLYSVLLD 45
ST46-2          MMQRSITQMSVTSQSCGMALSRMVQNTKTALGDFSFNSDIQDRRTFNTTETETLYSVLLD 60
ST46-1          MMQRSITQMSVTSQSCGMALSRMVQNTKTALGDFSFNSDIQDRRTFNTTETETLYSVLLD 60
ST76-1          MMQRSITPVSVTSQSCGMALSRMVQNTKTALGDFSFNSDIQDRRTFNTTETETLYSILLD 60
ST76-2          MMQRSITPVSVTSQSCGMALSRMVQNTKTALGDFSFNSDIQDRRTFNTTETETLYSILLD 60
                                 .  :   :..* ***************************:***

NTUH-P15        GKHSIAGTWEGELVRDNFAMTVKKSRGENRGVVITTHKNLKDYQRTKNSQNVVTRIHARS 105
ST46-2          GKHSIAGTWEGELVRDNFAMTVKKSRGENRGVVITTHKNLKDYQRTKNSQNVVTRIHARS 120
ST46-1          GKHSIAGTWEGELVRDNFAMTVKKSRGENRGVVITTHKNLKDYQRTKNSQNVVTRIHARS 120
ST76-1          GKHSIVGTWEGELVRDNFAITVKKSRGENRGVVITTHKNLKNYQRTKNSQNVVTRIHAKS 120
ST76-2          GKHSIVGTWEGELVRDNFAITVKKSRGENRGVVITTHKNLKNYQRTKNSQNVVTRIHAKS 120
                *****.*************:*********************:****************:*

NTUH-P15        TFKPEGAEKETTIRVTVDSPLINSYPYINEKEYENNNAKTVEELQKWAQSKFSNEGIDKV 165
ST46-2          TFKPEGAEKETTIRVTVDSPLINSYPYINEKEYENNNAKTVEELQKWAQSKFSNEGIDKV 180
ST46-1          TFKPEGAEKETTIRVTVDSPLINSYPYINEKEYENNNAKTVEELQKWAQSKFSNEGIDKV 180
ST76-1          TFKPEGAEKETTIRVTVDSPLINSYPYINEKEYENNNAKTVEELQKWAQSKFSNEGIDKV 180
ST76-2          TFKPEGAEKETTIRVTVDSPLINSYPYINEKEYENNNAKTVEELQKWAQSKFSNEGIDKV 180
                ************************************************************

NTUH-P15        SDAIKIEAYELDGQVVHMGDTANLKSWKHNVDVFKKAIAYEFDALKEEYISLTFNDKAGI 225
ST46-2          SDAIKIEAYELDGQVVHMGDTANLKSWKHNVDVFKKAIAYEFDALKEEYISLTFNDKAGI 240
ST46-1          SDAIKIEAYELDGQVVHMGDTANLKSWKHNVDVFKKAIAYEFDALKEEYISLTFNDKAGI 240
ST76-1          SDAIKIEAYELDGQVVHMGDTVNLKSWKHNVDAFKKAIAYEFDALKEEYISLTFDDKAGI 240
ST76-2          SDAIKIEAYELDGQVVHMGDTVNLKSWKHNVDAFKKAIAYEFDALKEEYISLTFDDKAGI 240
                *********************.**********.*********************:*****

NTUH-P15        GGSRASGGLSSAADAILGVTESAQEIALDKALQNADLDFDHKAGLLRQEISDGIELAKAR 285
ST46-2          GGSRASGGLSSAADAILGVTESAQEIALDKALQNADLDFDHKAGLLRQEISDGIELAKAR 300
ST46-1          GGSRASGGLSSAADAILGVTESAQEIALDKALQNADLDFDHKAGLLRQEISDGIELAKAR 300
ST76-1          GGSRASGGLSSAADAILGVTESAQEIALEKALQNADLDFDHKAGLLRQEISDDIELAKAK 300
ST76-2          GGSRASGGLSSAADAILGVTESAQEIALEKALQNADLDFDHKAGLLRQEISDDIELAKAK 300
                ***************************:**********************.********:

NTUH-P15        AEEVKRELSDTINQRFNSFDNGPLKETKRKAEEALRNAGASTLLAQEAKRIGLDSVARLE 345
ST46-2          AEEVKRELSDTINQRFNSFDNGPLKETKRKAEEALRNAGASTLLAQEAKRIGLDSVARLE 360
ST46-1          AEEVKRELSDTINQRFNSFDNGPLKETKRKAEEALRNAGASTLLAQEAKRIGLDSVARLE 360
ST76-1          AEEVKRELSDTINQRFNSFDNGPLKETKRKAEEALRNAGASSSLAQEAKRIGLDSVARLE 360
ST76-2          AEEVKRELSDTINQRFNSFDNGPLKETKRKAEEALRNAGASSSLAQEAKRIGLDSVARLE 360
                *****************************************: *****************
```

```
NTUH-P15    AFKSQTTSAQTALSGDLDALKRTIANDIRPKQAQAEAEIAKQVEALSRTKNELAGASSLL 405
ST46-2      AFKSQTTSAQTALSGDLDALKRTIANDIRPKQAQAEAEIAKQVEALSRTKNELAGASSLL 420
ST46-1      AFKSQTTSAQTALSGDLDALKRTIANDIRPKQAQAEAEIAKQVEALSRTKNELAGASSLL 420
ST76-1      AFKSQTTSAQTALSGDLDALKRTIVNDIRPKQAQVEAEIAKQAEALSRTKNELAG----- 415
ST76-2      AFKSQTTSAQTALSGDLDALKRTIVNDIRPKQAQVEAEIAKQAEALSRTKNELAG----- 415
            ************************.*********.*******.************

NTUH-P15    AQEAKRIELDSVARLEAFKSQTTSAQTALS------------------------------ 435
ST46-2      AQEAKRIELDSVARLEAFKSQTTSAQTALSGDLDVLKRTIANDIRPKQAQAEAEIAKQVE 480
ST46-1      AQEAKRIELDSVARLEAFKSQTTSAQTALS------------------------------ 450
ST76-1      ------------------------------------------------------------
ST76-2      ------------------------------------------------------------

NTUH-P15    ----------------------------------------------GDLDVLKRTIANDI 449
ST46-2      ALSRTKNELAGASTLLAQEAKRIELDSVARLEAFKSQTTSAQTALSGDLDVLKRTIANDI 540
ST46-1      ----------------------------------------------GDLDVLKRTIANDI 464
ST76-1      ------------------------------------------------------------
ST76-2      ------------------------------------------------------------

NTUH-P15    RPKQAQAEAEIAKQVEVLSRTKNELAGVKSAQATYEETTTRRLSELTNLANGKASKSELT 509
ST46-2      RPKQAQAEAEIAKQVEVLSRTKNELAGVKSAQATYEETTTRRLSELTNLANGKTSKSELT 600
ST46-1      PP---------------------------------------------------------- 466
ST76-1      ------------------------------------------------------------
ST76-2      ------------------------------------------------------------

NTUH-P15    QTAEELASRIASVQAGSSRNYFRNSRSRTFTTGGQAVYDYRTFIVPDFWKNSDRFKRDYV 569
ST46-2      QTAEELASRIASVQAGSSRNYFRNSRSRTFTTGGQAVYDYRTFIVPDFWKNSDRFKRDYV 660
ST46-1      -------------------NYFTNSRSRTFTTGGQAVYDYRTFIVPDFWKNSDRFKRDYV 507
ST76-1      ------------------------------------------------------------
ST76-2      ------------------------------------------------------------

NTUH-P15    RISFDVTFPVALVNDMPAMVHFSAHPWYAYRNLIFKGGTVERQHFEFTIDLSSSSEDYQT 629
ST46-2      RISFDVTFPVALVNDMPAMVHFSAHPWYAYRNLIFKGGTVERQHFEFTIDLSSSSEDYQT 720
ST46-1      RISFDVTFPVALVNDMPAMVHFSAHPWYAYRNLIFKGGTVERQHFEFTIDLSSSSEDYQT 567
ST76-1      ------------------------------------------------------------
ST76-2      ------------------------------------------------------------

NTUH-P15    NNVFIRFGTNYGFPAGLQVVIENAMLSVGNYFPAYQPAYEDQEDRVSVVESNFKQRADSL 689
ST46-2      NNVFIRFGTNYGFPAGLQVVIENAMLSVGNYFPAYQPAYEDQEDRVSVVESNFKQRADSL 780
ST46-1      NNVFIRFGTNYGFPAGLQVVIENAMLSVGNYFPAYQPAYEDQEDRVSVVESNFKQRADSL 627
ST76-1      ------------------------------------------------------------
ST76-2      ------------------------------------------------------------
```

```
NTUH-P15        DAGVSRLTEGLRTKADISALNVTAENIRQSVKSLETDTQNKLNQKLSQAEFEVRAGSIRQ 749
ST46-2          DAGVSRLTEGLRTKADISALNVTAENIRQSVKSLETDTQNKLNQKLSQAEFEVRAGSIRQ 840
ST46-1          DAGVSRLTEGLRTKADISALNVTAENIRQSVKSLETDTQNKLNQKLSQAEFEVRAGSIRQ 687
ST76-1          -----------------------------VKSAQATYKETTTRRLS-------------- 432
ST76-2          -----------------------------VKSAQATYKETTTRRLS-------------- 432
                                             *** ::  ::. .::**

NTUH-P15        EILNATKDKASKSELTQTAEELASRIASVQASGRNLFLNSLFKQDIPKTGIWTTSTYTAT 809
ST46-2          EILNATKDKASKSELTQTAEELASRIASVQASGRNLFLNSLFKQDIPKTGIWTTSTYTAT 900
ST46-1          EILNATKDKASKSELTQTAEELASRIASVQASGRNLFLNSLFKQDIPKTGIWTTSTYTAT 747
ST76-1          ELTNLANGKASKSELTQTAEELASRIASVQASGRNLFLNSLFKQDIPKTGIWTTSTYTAT 492
ST76-2          ELTNLANGKASKSELTQTAEELASRIASVQASGRNLFLNSLFKQDIPKTGIWTTSTYTAT 492
                *: * ::.****************************************************

NTUH-P15        IDSESKYLGHKALKIIGLNPSGRDGGNPKVTYPALGQFGKVIPGSTTNQDVTISFYAKAN 869
ST46-2          IDSESKYLGHKALKIIGLNPSGRDGGNPKVTYPALGQFGKVIPGSTTNQDVTISFYAKAN 960
ST46-1          IDSESKYLGHKALKIIGLNPSGRDGGNPKVTYPALGQFGKVIPGSTTNQDVTISFYAKAN 807
ST76-1          IDSESKYLGHKALKIIGLNPSGRDGGNPKVTYPALGQFGKVIPGSTTNQDVTISFYAKAN 552
ST76-2          IDSESKYLGHKALKIIGLNPSGRDGGNPKVTYPALGQFGKVIPGSTTNQDVTISFYAKAN 552
                ************************************************************

NTUH-P15        KNGIMLRSRLGNIGYKTGNVTLSTEIKRYVVHIPKGWTNESKQTTNEWLFNFNQEGTIWI 929
ST46-2          KNGIMLRSRLGNIGYKTGNVTLSTEIKRYVVHIPKGWTNESKQTTNEWLFNFNQEGTIWI 1020
ST46-1          KNGIMLRSRLGNIGYKTGNVTLSTEIKRYVVHIPKGWTNESKQTTNEWLFNFNQEGTIWI 867
ST76-1          KNGIMLRSRLGNIGYKTGNVTLSTEIKRYVVHIPKGWTNESKQTTNEWLFNFNQEGTIWI 612
ST76-2          KNGIMLRSRLGNIGYKTGNVTLSTEIKRYVVHIPKGWTNESKQTTNEWLFNFNQEGTIWI 612
                ************************************************************

NTUH-P15        WMPKFEISDVDTSYSEAPEDIEGQISTVESNFKQRADSLEAGVSRLTEGLRTKADISALN 989
ST46-2          WMPKFEISDVDTSYSEAPEDIEGQISTVESNFKQRADSLEAGVSRLTEGLRTKADISALN 1080
ST46-1          WMPKFEISDVDTSYSEAPEDIEGQISTVESNFKQRADSLEAGVSRLTEGLRTKADISALN 927
ST76-1          WMPKFEISDVDTSYSEAPEDIEGQISTVESTFKQRADSLDAGVRSLTEGLRTKADISSLN 672
ST76-2          WMPKFEISDVDTSYSEAPEDIEGQISTVESTFKQRADSLDAGVRSLTEGLRTKADISSLN 672
                ******************************.********:***  ***********:**

NTUH-P15        VTAENIRQSVKSLETDTQNKLNQKLSQAEFEVRAGSIRQEILNVTKDKASKSELTQTAEE 1049
ST46-2          VTAENIRQSVKSLETDTQNKLNQKLSQAEFEVRAGSIRQEILNVTKDKASKSELTQTAEE 1140
ST46-1          VTAENIRQSVKSLETDTQNKLNQKLSQAEFEVRAGSIRQEILNVTKDKASKSELTQTAEE 987
ST76-1          VTAENIRQSVKSLETDTQNKLNQKLSQAEFEVRAGSIRQEILNATKDKASKTELTQTAEE 732
ST76-2          VTAENIRQSVKSLETDTQNKLNQKLSQAEFEVRAGSIRQEILNATKDKASKSELTQTAEE 732
                *******************************************.*******:********
```

```
NTUH-P15      LSSKIASVQVGGINLLRNTASLLIGDRSK-GCWMSASGGNGRAISVEVLDPPKKMIKNMI 1108
ST46-2        LSSKIASVQVGGINLLRNTASLLIGDRSK-GCWMSASGGNGRAISVEVLDPPKKMIKNMI 1199
ST46-1        LSSKIASVQVGGINLLRNTASLLIGDRSK-GCWMSASGGNGRAISVEVLDPPKKMIKNMI 1046
ST76-1        LSSKIASVQASGRNLFLN--SLFKQDISKTGIWTTST--YTAAIDSESKYLGHKALKIIG 788
ST76-2        LSSKIASVQASGRNLFLN--SLFKQDISKTGIWTTST--YTAAIDSESKYLGHKALKIIG 788
             *********..* **: *  **:  * ** * * :::     **. *    :* :* :

NTUH-P15      RVIENTNGGNKDLTQLVRLRIGEKYTISCYARIASDSPNANVN---LLFRSWANNTDLNR 1165
ST46-2        RVIENTNGGNKDLTQLVRLRIGEKYTISCYARIASDSPNANVN---LLFRSWANNTDLNR 1256
ST46-1        RVIENTNGGNKDLTQLVRLRIGEKYTISCYARIASDSPNANVN---LLFRSWANNTDLNR 1103
ST76-1        LNPSGRDGGNPKVTYPALGQFGKVIPGSTTNQDVTISFYAKANKNGIMLRSRLGNIGYKT 848
ST76-2        LNPSGRDGGNPKVTYPALGQFGKVIPGSTTNQDVTISFYAKANKNGIMLRSRLGNIGYKT 848
                .. :*** .:*  .  ::*:  . *   : .: *  *:.*   :::**  .* . :

NTUH-P15      -KFQKSISHKNWQKYS---FTFTADAIENSIQFGQSGAGIIEICAPKIESGTLATDYSEA 1221
ST46-2        -KFQKSISHKNWQKYS---FTFTADAIENSIQFGQSGAGIIEICAPKIESGTLATDYSEA 1312
ST46-1        -KFQKSISHKNWQKYS---FTFTADAIENSIQFGQSGAGIIEICAPKIESGTLATDYSEA 1159
ST76-1        GNVTLSTEIKRYVVHIPKGWTNESKQTTNEWLFNFNQEGTIWIWMPKFEISDVDTSYSEA 908
ST76-2        GNVTLSTEIKRYVVHIPKGWTNESKQTTNEWLFNFNQEGTIWIWMPKFEISDVDTSYSEA 908
              :.  * . *.:  :    :*  :.   *.  *. .  * * *  **:* . : *.****

NTUH-P15      PEDIEGQISTVESTFKQRANSLDAGVSRLTEGLRTKVDISALNVTAENIRQSVKSLETDT 1281
ST46-2        PEDIEGQISTVESTFKQRANSLDAGVSRLTEGLRTKVDISALNVTAENIRQSVKSLETDT 1372
ST46-1        PEDIEGQISTVESTFKQRANSLDAGVSRLTEGLRTKVDISALNVTAENIRQSVKSLETDT 1219
ST76-1        PEDIEGQISTVESTFKQRANSLEAGVSRLTEGLRTKADISSLNVTAENIRQSVKSLETDT 968
ST76-2        PEDIEGQISTVESTFKQRANSLEAGVSRLTEGLRTKADISSLNVTAENIRQSVKSLETDT 968
             **********************:*************.***:*******************

NTUH-P15      QNKLNQKLSQAEFEVRAGSIRQEILNATKDKADKTLVVSEAGKLREEFSKMKVGGRNLWI 1341
ST46-2        QNKLNQKLSQAEFEVRAGSIRQEILNATKDKADKTLVVSEAGKLREEFSKMKVGGRNLWI 1432
ST46-1        QNKLNQKLSQAEFEVRAGSIRQEILNATKDKADKTLVVSEAGKLREEFSKMKVGGRNLWI 1279
ST76-1        QNKLNQKLSQAEFEVRAGSIRQEILNATKDKADKTLVVAEAGKLREEFSKMKVGGRNLWI 1028
ST76-2        QNKLNQKLSQAEFEVRAGSIRQEILNATKDKADKTLVVAEAGKLREEFSKMKVGGRNLWI 1028
             **************************************:*********************

NTUH-P15      KSKTVGAVIEKLPENHVTGQKECYRLENNSTLTFNLEPDFSSRLYQKVTFSAWIKYENVV 1401
ST46-2        KSKTVGAVIEKLPENHVTGQKECYRLENNSTLTFNLEPDFSSRLYQKVTFSAWIKYENVV 1492
ST46-1        KSKTVGAVIEKLPENHVTGQKECYRLENNSTLTFNLEPDFSSRLYQKVTFSAWIKYENVV 1339
ST76-1        KSKTVGAVIEKLPENHVTGQKECYRLENNSTLTFNLEPDFSSRLYQKVTFSAWIKYENVV 1088
ST76-2        KSKTVGAVIEKLPENHVTGQKECYRLENNSTLTFNLEPDFSSRLYQKVTFSAWIKYENVV 1088
             ************************************************************
```

```
NTUH-P15    QGRNFWNVFNCFKHYLFRKNSETGVQSGPDYATLGMYKGSADWKYITFTYDYSEKTNFDQ 1461
ST46-2      QGRNFWNVFNCFKHYLFRKNSETGVQSGPDYATLGMYKGSADWKYITFTYDYSEKTNFDQ 1552
ST46-1      QGRNFWNVFNCFKHYLFRKNSETGVQSGPDYATLGMYKGSADWKYITFTYDYSEKTNFDQ 1399
ST76-1      QGRNFWNVFNCFKHYLFRKNSETGVQSGPDYATLGMYKGSADWKYITFTYDYSEKTNFDQ 1148
ST76-2      QGRNFWNVFNCFKHYLFRKNSETGVQSGPDYATLGMYKGSADWKYITFTYDYSEKTNFDQ 1148
            ************************************************************

NTUH-P15    LKTSLRFNLEGATSGTAWVTGIKVEIGSVATDWSPAPEDADGLITEAKATFERTAQGLRT 1521
ST46-2      LKTSLRFNLEGATSGTAWVTGIKVEIGSVATDWSPAPEDADGLITEAKATFERTAQGLRT 1612
ST46-1      LKTSLRFNLEGATSGTAWVTGIKVEIGSVATDWSPAPEDADGLITEAKATFERTAQGLRT 1459
ST76-1      LKTSLRFNLEGATSGTAWVTGIKVEIGSVATDWSPAPEDADGLITEAKATFERTAQGLRT 1208
ST76-2      LKTSLRFNLEGATSGTAWVTGIKVEIGSVATDWSPAPEDADGLITEAKATFERTAQGLRT 1208
            ************************************************************

NTUH-P15    DLSAIQEYVNKNGQRQEALQRYTREESARQATAVRELVNRDFVGKATYQEDVKGINQRIE 1581
ST46-2      DLSAIQEYVNKNGQRQEALQRYTREESARQATAVRELVNRDFVGKATYQEDVKGINQRIE 1672
ST46-1      DLSAIQEYVNKNGQRQEALQRYTREESARQATAVRELVNRDFVGKATYQEDVKGINQRIE 1519
ST76-1      DLSAIQEYVNKDGQRQEALQRYTREESTRQATAVRELVNRDFVGKATYQEDVKGINQRIE 1268
ST76-2      DLSAIQEYVNKDGQRQEALQRYTREESTRQATAVRELVNRDFVGKATYQEDVKGINQRIE 1268
            ***********:***************:********************************

NTUH-P15    AVKTSANKDIASQIASYRQSVDGKFTDISSQITTYKQDVGGQISGLSNRLTSSEQGTTTQ 1641
ST46-2      AVKTSANKDIASQIASYRQSVDGKFTDISSQITTYKQDVGGQISGLSNRLTSSEQGTTTQ 1732
ST46-1      AVKTSANKDIASQIASYRQSVDGKFTDISSQITTYKQDVGGQISGLSNRLTSSEQGTTTQ 1579
ST76-1      AVKTSANKDIASQIASYRQSVDGKFTDISSQITTYKQDVGGQISGLSNRLTSSEQGTTTQ 1328
ST76-2      AVKTSANKDIASQIASYRQSVDGKFTDISSQITTYKQDVGGQISGLSNRLTSSEQGTTTQ 1328
            ************************************************************

NTUH-P15    ISNISNRINSNKQGTDNQISNLKTQVATNKDNAERQMGRISDQVSANKANADSQFANVTN 1701
ST46-2      ISNISNRINSNKQGTDNQISNLKTQVATNKDNAERQMGRISDQVSANKANADSQFANVTN 1792
ST46-1      ISNISNRINSNKQGTDNQISNLKTQVATNKDNAERQMGRISDQVSANKANADSQFANVTN 1639
ST76-1      ISNLSNRINSNKQGTDNQISNLKTQVATNKDNAERQMGRISDQVSANKANADSQFVNVTN 1388
ST76-2      ISNLSNRINSNKQGTDNQISNLKTQVATNKDNAERQMGRISDQVSANKANADSQFVNVTN 1388
            ***:***************************************************.****

NTUH-P15    QLARKVETTEFQRVKETSKLYERILGNTENGIADKVARMALTNQLFQVEVAKNASNGQNL 1761
ST46-2      QLARKVETTEFQRVKETSKLYERILGNTENGIADKVARMALTNQLFQVEVAKNASNGQNL 1852
ST46-1      QLARKVETTEFQRVKETSKLYERILGNTENGIADKVARMALTNQLFQVEVAKNASNGQNL 1699
ST76-1      QLARKVETTDFQRVKETSKLYERILGNTENGIADKVARMALTNQLFQVEVGKYSVSGPNL 1448
ST76-2      QLARKVETTDFQRVKETSKLYERILGNTENGIADKVARMALTNQLFQVEVGKYSVSGPNL 1448
            *********:****************************************.* : .* **
```

```
NTUH-P15      LKGTKDFSGGWKNKGANWKKHAEKYKGVDVLFKNNSWNGVGQEIDAKIGEVYTFSLWMKS 1821
ST46-2        LKGTKDFSGGWKNKGANWKKHAEKYKGVDVLFKNNSWNGVGQEIDAKIGEVYTFSLWMKS 1912
ST46-1        LKGTKDFSGGWKNKGANWKKHAEKYKGVDVLFKNNSWNGVGQEIDAKIGEVYTFSLWMKS 1759
ST76-1        IK-----NSDFKNATNEWGSTQNLGRLVKHSFYHNGQKDLMRLSNATKNENFLYSHRFNL 1503
ST76-2        IK-----NSDFKNATNEWGSTQNLGRLVKHSFYHNGQKDLMRLSNATKNENFLYSHRFNL 1503
             :*    ...:**   :* .  :  : *.  * :*. :.: :  :*. .* : :*  ::

NTUH-P15      DWKNDTAN---------------FYVNRNGSVEKGWGVPSETS---VAITSEWKRYSFTF 1863
ST46-2        DWKNDTVN---------------FYVNRNGSVEKGWGVPSETS---VAITSEWKRYSFTF 1954
ST46-1        DWKNDTVN---------------FYVNRNGSVEKGWGVPSETS---VAITSEWKRYSFTF 1801
ST76-1        ERNTDYVLNFRGFNNSALASYDVYILGRRAGESDGFTIVKKVVSSKKLSTSRCEDVSVTF 1563
ST76-2        ERNTDYVLNFRGFNNSALASYDVYILGRRAGESDGFTIVKKVVSSKKLSTSRCEDVSVTF 1563
             : :.* .              : :.*... ..*: : .:.      **. :  *.**

NTUH-P15      KITVDGFIFPRVERLNQN---TNLYIAGLKLEKGSYATPYTEAPEDTD-------EAIRS 1913
ST46-2        KITVDGFIFPRVERLNQN---TNLYIAGLKLEKGSYATPYTEAPEDTD-------EAIRS 2004
ST46-1        KITVDGFIFPRVERLNQN---TNLYIAGLKLEKGSYATPYTEAPEDTD-------EAIRS 1851
ST76-1        NSGEMDNAYIRFDNNGSSSGTADLYITEVDLYKGYKPRTWQPHPEDVVADANKKLEATQT 1623
ST76-2        NSGEMDNAYIRFDNNGSSSGTADLYITEVDLYKGYKPRTWQPHPEDVVADANKKLEATQT 1623
             :    .  : *.:. ...  ::***: :.* **  . .:   ***.       ** ::

NTUH-P15      VQSQLTGSWAVQNINSAGDIISGINLGANGHNRFVGKLTHITGETLIDRAVIKSAMVDKL 1973
ST46-2        VQSQLTGSWAVQNINSAGDIISGINLGANGHNRFVGKLTHITGETLIDRAVIKSAMVDKL 2064
ST46-1        VQSQLTGSWAVQNINSAGDIISGINLGANGHNRFVGKLTHITGETLIDRAVIKSAMVDKL 1911
ST76-1        KMTLLTGSWAVQNINSAGDIISGINLGANGHNRFVGKLTHITGETLIDRAVIKSAMVDKL 1683
ST76-2        KMTLLTGSWAVQNINSAGDIISGINLGANGHNRFVGKLTHITGETLIDRAVIKSAMVDKL 1683
               : ********************************************************

NTUH-P15      KTA--------------------ILKLVRSRLRY--------------- 1987
ST46-2        KTANFEAGSVTTTTILDAEAVTAEKLKVDNALIRKLTATDAF-------- 2105
ST46-1        KTANFEAGSVTTTTILDAEAVTAEKLKVDNALIRKLTATDAFIDQLISKT 1960
ST76-1        KTGNFEAGSVTTTTILDAEAVTAEKLKVDNALIRKLTANDAFI------- 1725
ST76-2        KTGNFEAGSVTTTTILDAEAVTAEKLKVDNALIRKLTANDAFI------- 1725
             **.                 **: .: :*
```

Supplementary Figure S1. Comparison of amino acid sequences of PblB in NTUH-P15, serotype 14 ST46-1, serotype 14 ST46-2, serotype 6B ST76-1 and serotype 6B ST76-2. Gray shaded area indicted predicted galactose-binding domain-like region.
